# Enabling Breeding Selection for Biomass in Slash Pine Using UAV-Based Imaging

**DOI:** 10.34133/2022/9783785

**Published:** 2022-04-22

**Authors:** Zhaoying Song, Federico Tomasetto, Xiaoyun Niu, Wei Qi Yan, Jingmin Jiang, Yanjie Li

**Affiliations:** ^1^Research Institute of Subtropical Forestry, Chinese Academy of Forestry, No. 73, Daqiao Road, Fuyang, Hangzhou, 311400 Zhejiang Province, China; ^2^College of Landscape and Travel, Agricultural University of Hebei, Baoding, China; ^3^AgResearch Ltd., Christchurch 8140, New Zealand; ^4^Auckland University of Technology, Auckland 1010, New Zealand

## Abstract

Traditional methods used to monitor the aboveground biomass (AGB) and belowground biomass (BGB) of slash pine (*Pinus elliottii*) rely on on-ground measurements, which are time- and cost-consuming and suited only for small spatial scales. In this paper, we successfully applied unmanned aerial vehicle (UAV) integrated with structure from motion (UAV-SfM) data to estimate the tree height, crown area (CA), AGB, and BGB of slash pine for in slash pine breeding plantations sites. The CA of each tree was segmented by using marker-controlled watershed segmentation with a treetop and a set of minimum three meters heights. Moreover, the genetic variation of these traits has been analyzed and employed to estimate heritability (*h*^2^). The results showed a promising correlation between UAV and ground truth data with a range of *R*^2^ from 0.58 to 0.85 at 70 m flying heights and a moderate estimate of *h*^2^ for all traits ranges from 0.13 to 0.47, where site influenced the *h*^2^ value of slash pine trees, where *h*^2^ in site 1 ranged from 0.13~0.25 lower than that in site 2 (range: 0.38~0.47). Similar genetic gains were obtained with both UAV and ground truth data; thus, breeding selection is still possible. The method described in this paper provides faster, more high-throughput, and more cost-effective UAV-SfM surveys to monitor a larger area of breeding plantations than traditional ground surveys while maintaining data accuracy.

## 1. Introduction

Carbon emissions refer to greenhouse gas emissions that contribute to the greenhouse effect and global temperature rise. The largest proportion of greenhouse gases is composed by CO_2_ and is the concentration of CO_2_ in the atmosphere increases and it becomes the main causal factor for one of the most vital issues in the twenty-first century, i.e., global warming [1]. One solution to reduce global warming is that carbon needs to be sequestered from the atmosphere. Trees are the simplest, most natural, and environmentally friendly way for carbon sequestration [2]. It has been reported that forests can capture 14.1 PgC yr^−1^ of CO_2_ through photosynthesis while releasing 11.6 PgC yr^−1^ of CO_2_ through respiration yielding a positive capture and storage balance [3]. However, different type of forests could result in varying abilities of CO_2_ sequestration by forests. Therefore, in recent years, the ability of trees and forests to absorb CO_2_ emissions and mitigate climate change has been of a global concern [4, 5].

Slash pine (*Pinus elliottii*) is native to the southeastern United States. With its excellent characteristics, such as rapid growth, strong adaptability, and high yield of resin, slash pine has been successfully introduced to southern China since 1930s for afforestation [6, 7]. At present, slash pine plantations have reached over 3 million hectares in subtropical areas and have become one of the largest timber and resin production suppliers in China [8]. Pine plantations have been suggested to be an important part of global carbon sequestration both through the accumulation of carbon (C) in wood for long-lasting products together with an increase in resin yield [6, 9–11]. In addition, moderate resin-tapping intensity in slash pine plantations creates an increased carbon sink and does not cause a limitation in wood carbon allocation for growth [12]. Therefore, slash pine is one of the most important carbon sink tree species due to its ability to store large amounts of carbon in its living biomass.

Individual tree biomass monitoring and measurement have traditionally relied on difficult and costly ground surveys for collecting tree inventory data that involve destructive sampling and cover relatively small spatial scales (several hectares). Therefore, any high-throughput and nondestructive measurements, as a potential biomass indicator that can easily be applied to biomass crops, would be advantageous. So far, there are two indirect approaches for tree biomass measurement, including biomass factor methods and biomass models [13]. Forest inventory information (i.e., diameter, height, or volume data), as well as various factors alone or in combination with other factors (i.e., vegetation indices), can be deployed to estimate biomass. High-accuracy estimation of forest carbon storage requires large-scale geographic region inventory data. Therefore, a national program to estimate individual tree biomass across China was launched in 2009 [14], which included a special tree biomass model for slash pine. Based on the tree height and diameter at breast height (DBH), the individual tree aboveground biomass (AGB) and belowground biomass (BGB) of slash pine were successfully predicted with mean prediction errors of less than 5% and 7.5%, respectively [15].

The growth traits and biomass of trees vary with and within families [16]. Breeding is able to exploit these variations for optimal trait selection [17]. Biomass, especially AGB, has been identified as the most important trait to exploit in tree breeding programs for the high production of biomass plantations to mitigate the effects of climate change. Genetic variations in growth traits, adaptability, resin, and wood productivity in slash pine have been extensively reported [18–21].

One of the most important steps in breeding programs is the transformation in detail and speed of genetic information that is then brought to the next generation [22]. Traditionally, tree height and diameter are measured manually with graduated poles and measuring tapes, which is time- and labor-consuming and difficult for tall trees in complex terrains [23]. Remote sensing is an efficient technique for measuring tree crops and has the capability of providing multitemporal information on tree structure. Recently, unmanned aerial vehicles (UAVs) equipped with LiDAR or RGB imagery have been demonstrated to work very well in forestry [24–26]. Compared to RGB imagery, UAV-based LiDAR holds the advantage of obtaining point cloud data and achieves relatively high accuracy [27]. Still, LiDAR equipment is very expensive and has limitations in low efficiency and complicated operations, making it unsuitable for routine operations in a large-scale forest [28]. Alternatively, a low-cost UAV-based RGB digital camera equipped with a real-time kinematic positioning system (RTK) can also generate 3D point cloud data with high accuracy in a relatively sparse forest [29, 30]. UAV-based RGB imagery has been successfully applied to numerous conifer forests for tree growth measurements [30, 31]. Previously, study has been shown that UAV-multispectral platforms could serve as an rapid method for breeding selection of vegetation indices in slash pine trees [32]. However, prior to this project, UAV-based RGB imagery has not been employed in slash pine plantations for breeding purposes.

UAV-based RGB imagery with an RTK system is able to obtain photogrammetric images by overlapping photographs with the SfM method to produce 3D georeferenced points, which are further separated into a digital terrain model (DTM) and a digital surface model (DSM) for crop height model (CHM) computation. Further analysis of the CHM allows the estimation of tree height, DBH, CA, and biomass to be employed in breeding programs [33]. Flight altitude influences the accuracy of UAV-based photogrammetric data if other factors, such as the UAV platform (multirotor fixed-wing), camera specification, and air conditions, are fixed. High altitude requires much less capture time than low altitude scarifying image resolution and resulting in a low density of point clouds [34]. Therefore, how the changes in UAV flying altitude influence the quality of 3D georeferenced points should be clarified.

Hence, this study evaluated the use of UAV-based images in a slash breeding plantation with four main objectives: (1) to assess tree heights and CA from the CHM generated with images captured from a low-cost high-resolution camera; (2) to determine the influence of different flying altitudes on the 3D georeferenced point cloud quality and its accuracy of tree height and CA estimations; (3) to estimate the DBH and biomass (AGB and BGB) by combining CHM-based height and CA data; and (4) to compare the genetic gains from UAV and ground truth data to demonstrate the capabilities of UAV-obtained biomass for slash pine breeding.

## 2. Materials and Methods

### 2.1. Study Area and Tree Materials

The experiment was carried out based on slash pine progeny trials in the Matou National Forest Farm in Xuancheng, Anhui, China (30° 45′ N, 118° 29′ E). This region has a subtropical temperate monsoon humid climate, and the average temperature and precipitation are 15.7°C and 1,520 mm annually, respectively. Seeds from 20 open-pollinated families were collected in 2011 and planted in a nursery. In 2013, 1-year-old seedlings were planted systematically using an alpha lattice incomplete block and single-tree-plot design at two sites. Each block contained 20 trees with a 2 m × 3 m spacing. Each tree represented one family, with no repeated family within a block. In total, there were 560 individual trees. The altitude in site 1 is ~10 m higher than site 2p; there are 257 and 303 living trees in site 1 and site 2, respectively.

### 2.2. UAV Image Acquisition and Field Data

**Figure 1:**
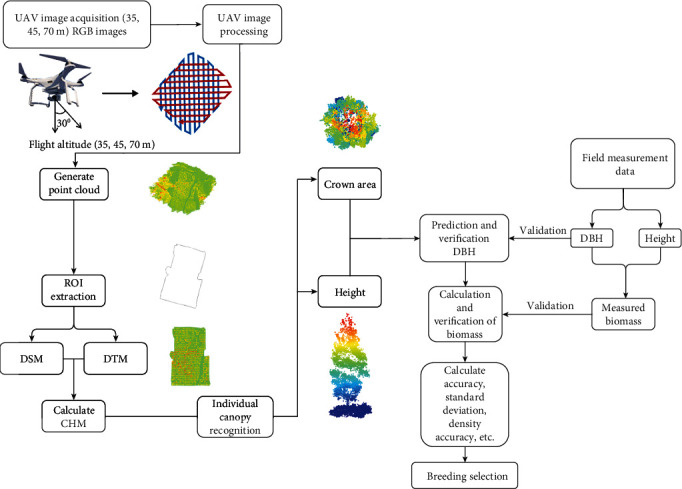
Workflow used to evaluate the potential usage of UAV-SfM-based methods to estimate above- and belowground biomass of slash pine breeding plantations at Matou National Forest Farm, Jing County, Xuancheng City, Anhui Province, China.

A low-cost UAV with an in-built high-resolution RGB sensor was employed for individual tree structural characteristic detection and measurement of slash pine in China. Meanwhile, the ground truth tree height and DBH data were measured for validation. Details of the workflow can be found in Figure 1.

During a sunny day without clouds and light winds on 12 July 2021, three completed cross-hatched flights were performed at 35 m, 45 m, and 70 m altitudes, respectively, using the low-cost drone DJI Phantom 4 RTK (DJI, Shenzhen, Guangdong, China) equipped with a high-definition camera (20 million pixels). Coordinate correction via the network RTK (NRTK) service was deployed. The horizontal and vertical positioning errors were 0.03 m and 0.06 m, respectively. The sensor captured images in jpeg format; image dimensions were set to 3 : 2, and the overhead trajectory of UAV operation had an 80% longitudinal and lateral overlap to meet the accuracy requirements. DJI GS Pro software (version 2.0.15; Shenzhen, China) for Apple iOS was used for all image collection. In total, 2,251 images covering an area of 41,000 m^2^ were collected with a 2.5 h in-air flight time. The flight settings are shown in Table 1. Based on the map of the breeding plantation and for ground-truth purposes, we randomly selected 100 trees to measure tree height and DBH and recorded the tree site, block, and family information.

### 2.3. UAV Image Processing

The original raw images with georeferenced information were analyzed through structure from motion (SfM) photogrammetry to generate georeferenced 3D dense cloud points of the slash plantation at three altitudes in DJI Terra software (version 3.0.4, Shenzhen, China). The overall methodology involved was canopy height model (CHM) 3D point clouds and 2D raster images were normalized by subtracting the DTM from the original point cloud and then analyzed to detect individual tree locations, crown areas, and heights using a local maximum variable window function [33]. The three-dimensional reconstruction clarification quality was set as high as possible. Finally, the 3D cloud points were exported as LAS files and used in R version 4.0.1 software [35] for further processing. The *X*-, *Y*-, and *Z*-coordinate attributes of the cloud points were loaded in R software and used to process the classification of ground points using Cloth Simulation Filter (CSF) algorithms [36] in the *lidR* package [37]. Then, based on the classification of ground points and the spatial interpolation algorithm (*k*-nearest neighbor (KNN) approach with inverse-distance weighting (IDW)), digital terrain models (DTMs) with a 0.5 m resolution at three different altitudes were generated. Subsequently, each CHM was investigated to identify the local maximum heights by using a variable diameter of the moving window (Equation (1)) to assign the locations (*x* and *y* coordinates), CA, and minimum height (m) of the tree >2.6 m.

(1) $$\begin{array}{c} Vwr=H\times 0.05+2.6, \end{array}$$

where *Vwr* refers to the variable window radius and *H* is the tree height.

The *dalponte2016* function [38] in the *lidR* package was deployed for individual tree segmentation and polygons generation. This method is mainly based on the treetops with a minimum height of 2.6 m and a maximum crown diameter of 5 m. These parameters were chosen to avoid confusion with neighboring trees when running segmentation ensuring that all of the trees have been detected. As a postprocessing step, the detected individual trees with an ID number were converted to a 2D convex hull to generate tree crown polygons; tree height and crown area information of each individual tree were extracted using *raster* [39] and tidyverse packages [40]. Each tree with ID number was manually matched with the site, block, and family information.

### 2.4. Statistical Analysis

#### 2.4.1. DBH Prediction Model

DBH has a strong positive correlation with tree height and CA [41]. Therefore, it is possible to use tree height and CA as predictor variables for estimating DBH. Since the relationship among DBH, tree height, and CA does not exactly follow a linear correlation [42], a nonlinear generalized additive model (GAM) [43] has been applied [44, 45]. The measured ground truth of 100 tree DBH and height data was used for validation. The GAM equation is

(2) $$\begin{array}{c} \text{DBH}=a+f_{1\left({\text{Height}}_{i}\right)}+f_{2\left({CA}_{i}\right)}+\varepsilon , \end{array}$$

where DBH is the response variables, *a* is the intercept, *f*_1_ and *f*_2_ are smooth functions of covariates for the *i*_th_ independent of height and CA variables, and *ε* is a vector of unobserved random errors. The restricted maximum likelihood (REML) was taken as the smoothing parameter estimation method.

#### 2.4.2. AGB and BGB Calculation

The estimation of AGB (kg) and BGB (kg) for slash pine presented by Fu et al. [15] was adopted in this paper. The equations are listed as follows:

(3) $$\begin{array}{c} M_{a}=0.0861D^{2.072}H^{0.452}+0.002, \end{array}$$

(4) $$\begin{array}{c} M_{b}=0.0269D^{2.394}H^{0}+0.058, \end{array}$$

where *M*_*a*_ and *M*_*b*_ are the aboveground and belowground biomass, respectively, and *D* and *H* are the tree DBH and height, respectively.

#### 2.4.3. Estimation of Genetic Parameters

A restricted maximum likelihood (REML) bivariate linear mixed model was fitted for tree growth and biomass generated from UAVs to estimate the genetic parameters for breeding evaluation. Details have been reported by Li et al. [46]. Briefly, model (5) is shown as:

(5) $$\begin{array}{c} \text{y}=Xm+Z_{1}b+Z_{2}f+e, \end{array}$$

where *y* represents the response vector of tree traits and *X*, *Z*_1_, and *Z*_2_ are the incidence matrices linking observations to the appropriate effects. Consequently, *m*, *b*, *f*, and *e* are the vectors of intercept, site effects, and the vectors of random additive effects for block, family, and residual effects, respectively. We used variance components from the model to estimate the narrow sense of *h*^2^ and genetic correlation (*r*_*gij*_) between each trait. At each site, breeding values were considered to calculate the genetic gain (*Δ*GR) by subtracting the mean breeding values of selected top ratio tree growth traits from the total mean of the tree growth trait and subsequently estimating the difference between them. Equation (6) and Equation (7) show the relationship between *h*^2^ and *r*_*gij*_.

(6) $$\begin{array}{c} h_{i}^{2}=\frac{2.5\sigma_{fi}^{2}}{\sigma_{f_{i}}^{2}+\sigma_{b_{i}}^{2}+\sigma_{e_{i}}^{2}}, \end{array}$$

(7) $$\begin{array}{c} r_{gij}=\frac{\sigma_{fij}}{\sqrt{\sigma_{f_{i}}^{2}\sigma_{f_{j}}^{2}}}, \end{array}$$

where *σ*_*fi*_^2^, *σ*_*b*_*i*__^2^, and *σ*_*e*_*i*__^2^ are the family, block, and residual variance for trait *i*, respectively. *σ*_*f*_*j*__^2^ is the family variance for trait *j*, and *σ*_*fij*_ is the estimated family covariance between trait *i* and trait *j*.

All analyses were accomplished with R software by using the RStudio platform [47]. The *gam* package [48] was used for GAM model fitting, the *sommer* package [49] was employed for genetic variance and covariance estimations, and the *ggplot2* package [50] was harnessed for visualization.

## 3. Results

### 3.1. Canopy Height Model Computation and Individual Tree Detection

**Figure 2:**
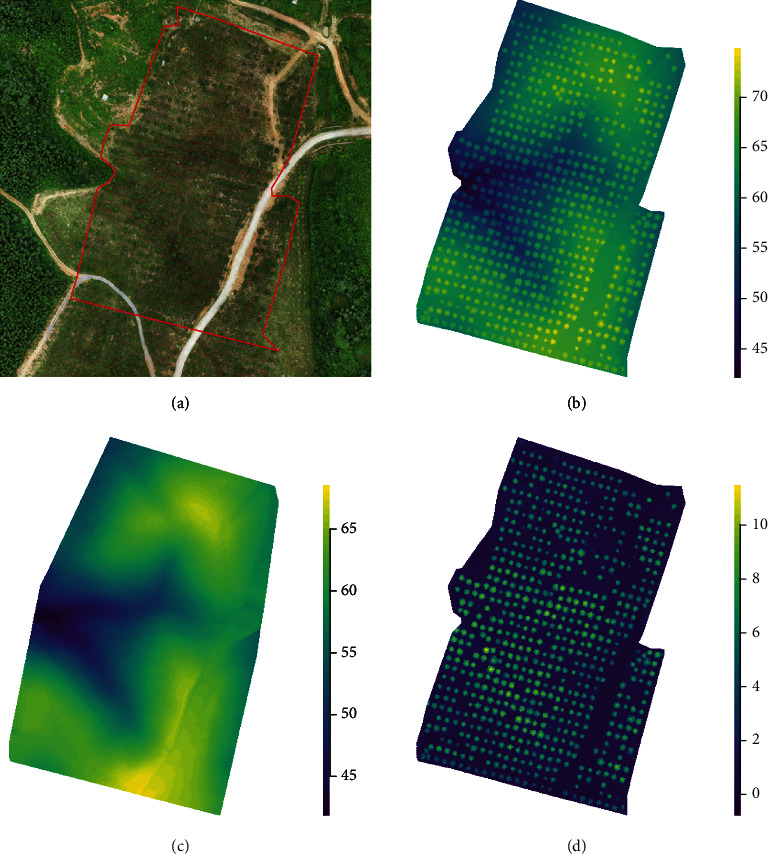
Images of the study area derived from UAV data: (a) orthomosaic image; (b) digital surface model; (c) digital terrain model; and (d) canopy height model.

**Figure 3:**
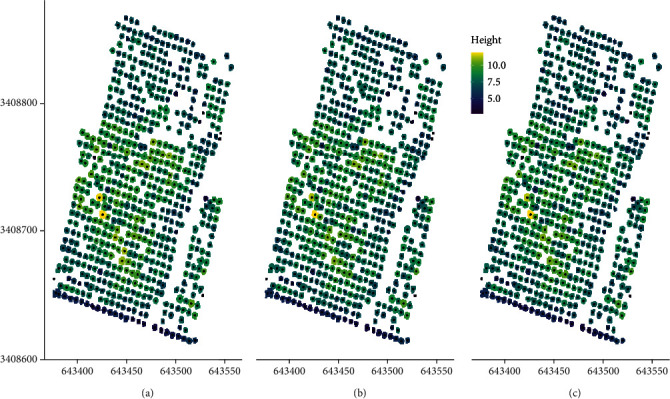
The heterogeneous profile of heights from three different flight altitudes (30 m(a), 45 m(b), and 70 m(c)) measured using the UAV-SfM method. The canopy height model (CHM) shows individual treetop locations (black dots) and associated tree crown perimeter (colored area). Different colors represent different tree heights.

The examples of RGB orthomosaics, DSMs, DTMs, and CHMs generated from 3D point clouds from UAV-SfM at 70 m altitude flight are shown in Figure 2 with an average pixel resolution of 0.5 m. The tree UAV_CA and treetop of all of the target trees from the two sites were successfully delimited at flight altitudes of 35, 45, and 70 m (Figure 3).

Comparing images of the point cloud during different collection flight altitudes, the number of point cloud at 45 m is significantly larger than the point cloud density generated at 35 m and 70 m (Figure S1). Trees grew better in the low-altitude area than in the higher-altitude area (Figure 3). However, tree height, UAV_CA, and biomass (UAV_AGB and UAV_BGB) data generated from CHMs at different flight altitudes did not show significant differences (Table 2 and Figure S1).

### 3.2. Tree Growth Traits and Biomass (AGB and BGB) Estimation Assessment

**Figure 4:**
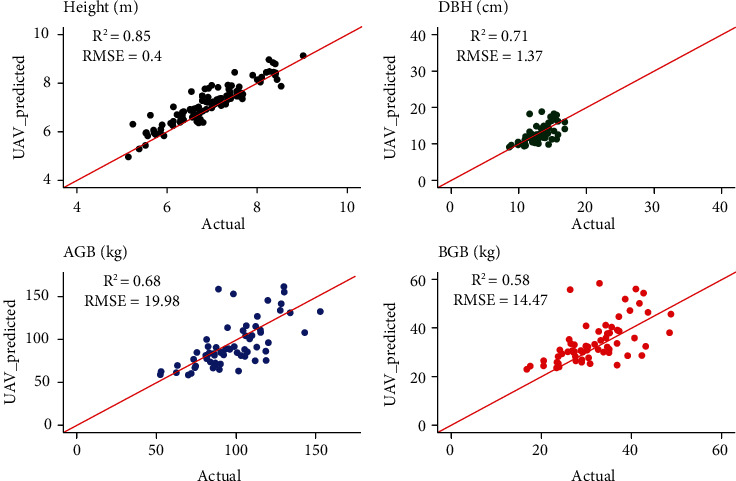
The linear model and the ground truth data, UAV data (height, AGB, and BGB) and predicted DBH at 70 m flying heights.

**Figure 5:**
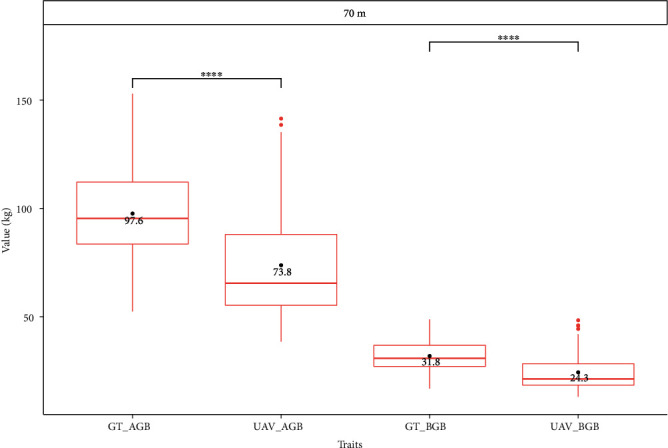
Significant differences between ground truth and UAV-generated aboveground and belowground biomass from 70 m flight altitudes. UAV_AGB: UAV-generated aboveground biomass; UAV_BGB: UAV-generated belowground biomass; GT_AGB: ground truth aboveground biomass; GT_BGB: ground truth belowground biomass; ^∗∗∗^*p* ≤ 0.001, ∗∗∗∗*p* ≤ 0.0001.

The relationship between predicted and measured values of traits such as actual ground truth (GT) and UAV-SfM (UAV) height, DBH, AGB, and BGB values generated at 70 m flight altitude showed relatively promising results (Figure 4 and Figure S2). The tree heights measured and predicted at flight altitudes of 35, 45, and 70 m exhibited the highest *R*^2^ values of 0.85, 0.86, and 0.85, respectively, with the lowest RMSEs of 0.36, 0.4, and 0.4, respectively (Figure S2), followed by the AGB, DBH, and BGB traits. The estimated AGB and BGB based on the UAV-SfM data in the breeding plantations were consistently lower (~30%) than the values generated from the ground truth data (Figure 5).

### 3.3. Family Ranking and Heritability of Growth Traits and Biomass Properties at Two Sites

**Figure 6:**
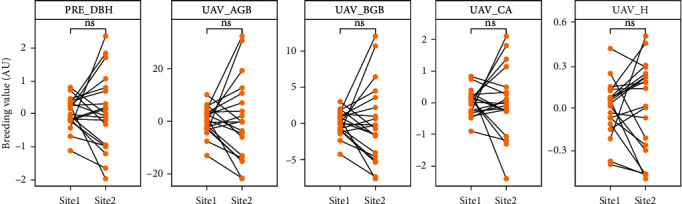
Visualization of differences in breeding value for slash pine for each trait (UAV_H, PRE_DBH, UAV_CA, UAV_AGB, and UAV_BGB and genetic correlation) between two different sites, ns: no significance (*p* > 0.05).

**Figure 7:**
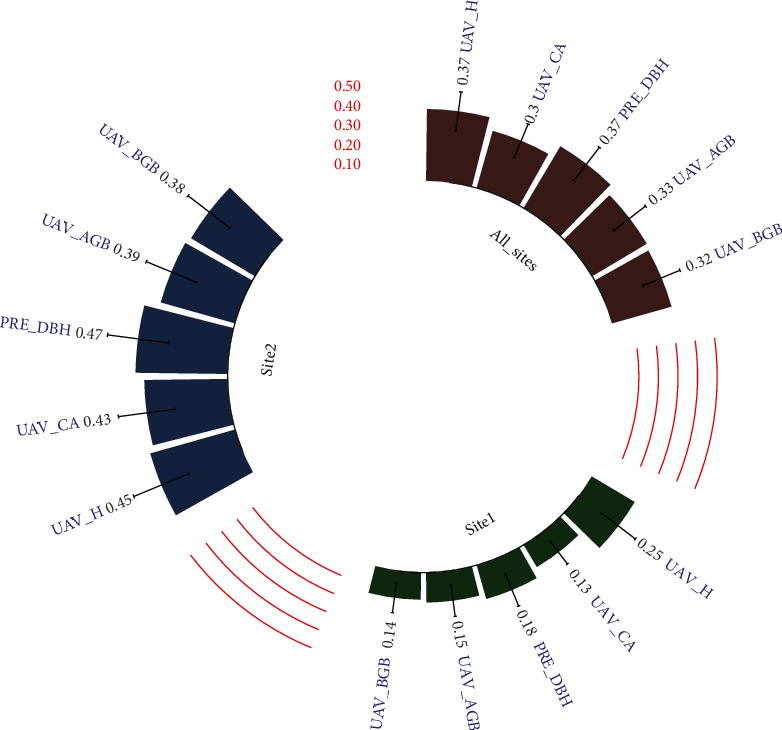
Estimates of *h*^2^ for growth and biomass traits at two sites.

The predicted DBH (PRE_DBH), crown area (UAV_CA), tree height (UAV_H), aboveground biomass (UAV_AGB), and belowground biomass (UAV_BGB) generated from 70 m altitude UAV-SfM data were considered for the final genetic variation analysis. Most of the families showed to have consistent rankings in all traits for the two sites, which indicates that it is possible to select families with good growth and biomass traits using genetic selection (Figure 6). The heritability (*h*^2^) of all traits at each site and their combined yield ranged from 0.13 to 0.47. The total *h*^2^ for all sites generated a promising *h*^2^ for all traits, with a range from 0.3 to 0.37. All traits generated lower *h*^2^ values of all traits (range: 0.13-0.25) at site 1 than at site 2 (range: 0.38-0.47). The highest *h*^2^ values at site 1, site 2, and all sites combined were for UAV_H (*h*^2^ = 0.45), PRE_DBH (*h*^2^ = 0.47), UAV_AGB (*h*^2^ = 0.39), UAV_BGB (*h*^2^ = 0.38), and UAV_CA (*h*^2^ = 0.43), respectively. The UAV_BGB generates lower heritability at site 1, site 2, and all sites combined (Figure 7).

### 3.4. Genetic Correlations between Traits and Family Selection

**Figure 8:**
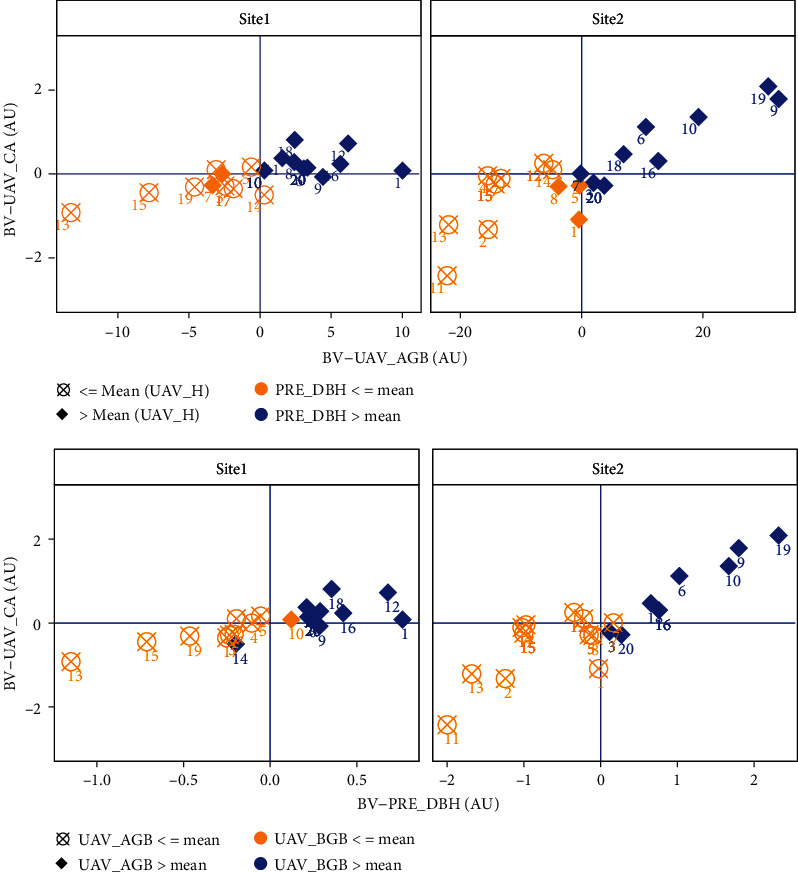
The correlation between DBH and the genetically related breeding values of the UAV predicted height, AGB, and BGB at the two different sites. BA-UAV_CA: breeding value of crown area; BA-PRE_DBH: breeding value of diameter at breast height; BA-UAV_AGB: breeding value of aboveground biomass.

Table 3 shows the estimated genetic correlations between different traits at site 1 and site 2. All traits showed a high genetic correlation at both sites. The GT_AGB and GT_BGB have a significant genetic correlation with UAV_AGB and UAV_BGB in both sites, with a range of *r*_*g*_ from 0.63 to 0.93. The UAV_AGB and UAV_BGB have high genetic correlations with UAV_H (*r*_*g*_ = 0.99 and 0.99 at site 1 and *r*_*g*_ = 0.79 and 0.75 at site 2, respectively) and PRE_DBH (*r*_*g*_ = 0.97 and 0.96 at site 1 and *r*_*g*_ = 0.98 and 0.96 at site 2, respectively). Genetic correlations were moderately high between UAV_CA and UAV_H (*r*_*g*_ = 0.77 at site 1 and *r*_*g*_ = 0.68 at site 2). High positive genetic correlation indicates that there is potential for selecting families with multiple optimal traits together. According to different breeding goals, families with optimal breeding values of multiple traits may be selected. The UAV_AGB and UAV_BGB have a significant positive genetic correlation (1 in all sites). Therefore, the large CA and high AGB and BGB are shown in Figure 8, and the breeding values that are higher than the mean UAV_AGB and UAV_CA are shown in the first quadrant. In addition, optimal families with high UAV_H, PRE_DBH, UAV_AGB, UAV_BGB, and UAV_CA were found, including families 1, 6, 8, 9, 11, 12, 16, 18, and 20 at site 1 and families 6, 9, 10, 16, 18, and 19 at site 2, in which families 6, 9, 16, and 18 show high breeding values at both sites.

### 3.5. Genetic Gains

**Figure 9:**
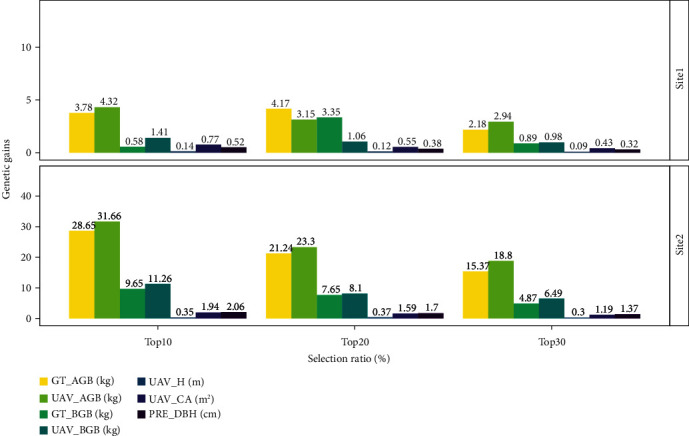
Realized genetic gains of growth and biomass traits at age 8 for slash pine at the two sites. UAV_AGB: UAV-generated aboveground biomass; UAV_BGB: UAV-generated belowground biomass; GT_AGB: ground truth aboveground biomass; UAV_CA: UAV-generated crown area; UAV_H: UAV-generated tree height; PRE_DBH: predicted DBH; GT_BGB: ground truth belowground biomass.

The realized genetic gains were calculated by selecting the top 10, 20, and 30% of the families for each trait (Figure 9). All traits showed an overall higher genetic gains at site 2 than at site 1. In particular, the UAV_AGB and UAV_BGB genetic gains at site 2 were ~5 times higher than the genetic gains at site 1. The genetic gain of ground truth AGB and BGB are similar to the UAV generated AGB and BGB in both sites, respectively.

## 4. Discussion

To the best of our knowledge, limited research regarding the prediction of slash pine attributes for breeding selection using remote sensing techniques is available. First, we demonstrated the ability of UAV-based imagery (in this case UAV-SfM data) to estimate tree growth traits, AGB, and BGB of slash pine in a breeding plantation [30, 51]. In this context and regarding LiDAR technology [52, 53], Kuyah et al. [54] found that UAV-derived LiDAR yields are slightly better estimated than yields from UAV imagery for tree height measurements, but the cost of LiDAR is much higher than UAV imagery [55]. Therefore, in this study, low-cost UAV-based imagery was chosen for the creation of orthomosaic images to generate CHMs for slash pine plantations at three flight altitudes. Furthermore, a high correlation was found between the UAV-SfM estimated and the ground truth measures of tree height, DBH, CA, AGB, and BGB.

### 4.1. Comparison between UAV-SfM and Field Data-Derived Metrics

The flight altitudes of 35 m, 45 m, and 70 m generated tree growth traits with relatively high prediction accuracy and did not show a significant difference based on the tree growth traits, AGB and BGB yields. However, the flight time at 70 m is significantly less than the flight time at the other two altitudes, with only 17.56 minutes compared to 45.35 minutes at 35 m and 42.47 minutes at 45 m (Table 1). Similar results were reported by Avtar et al. [56], who found that (1) the CA derived at flight altitudes of 20 m, 60 m, and 80 m yields a similar correlation with the ground truth of young oil palm (*Elaeis guineensis*); (2) tree heights derived from 60 m and 80 m flights showed slightly higher correlations with ground truth data than those derived from the 20 m flight altitude. Moreover, Sadeghi and Sohrabi [57] found that a higher flight altitude (80-140 m) yielded more accurate height measurements than a lower flight altitude. Surový et al. [58] also found that higher flight altitude indicates better forestry inventory results and requires less time than a lower flight altitude for the collection of image data and computer processing.

The manual assessment of tree heights, CA, and DBH is time-consuming and suitable for only a small portion of forest fields. Here, the high-throughput phenotyping method UAV-based imaging was applied to estimate the canopy height and CA. All 560 trees in the field were detected using the UAV-SfM methods. This value is more accurate than the values of other studies with the same methods for tree detection from SfM-derived products in high-tree density forests [59, 60]. This high level of estimation is highly accepted for forestry and biomass inventories in plantations with low tree densities when only the tree height and CA are considered.

In this study, the UAV-SfM-derived CHMs was highly correlated with the ground truth measurements (Figure 4). With the onboard RTK systems, the UAV-SfM data yielded reliable results when generating DTM and DSM, which resulted in a high accuracy of tree height, with *R*^2^ and RMSE values ranging from 0.85 to 0.86 and 0.36 to 0.40 m, respectively. UAV-SfM has also been successfully applied to other canopy height measurements, such as *Eucalyptus* [61], *Pinus pinea* [62], and oil palm (*Elaeis guineensis*) [63] measurements, and has achieved high accuracy, with an *R*^2^ larger than 0.8.

In our study, the DBH prediction was less accurate, with a mean *R*^2^ and RMSE of 0.64 and 2.60 cm, respectively, from three flight altitudes. These results are consistent with the study reported by Zhou et al. [64], who found that the highest *R*^2^ and RMSE for DBH were 0.66 and 1.97 cm, respectively, when using 12 traditional DBH estimation models. The UAV-SfM technology can be easily used to detect the tree height and crown, but can rarely be used to derive the DBH [40]. The less accuracy of DBH estimations highly affects the prediction of AGB and BGB yields, which leads to a mean *R*^2^ and RMSE being 0.69 and 19.70 kg for AGB and 0.59 and 14.26 kg for BGB, respectively, Those results were also similar to Jones et al. [51], who also found that UAV image-based measurements provide poor prediction of DBH and consistently affected the estimate of biomass yields. Our results showed that UAV-SfM-estimated AGB is approximately 30% lower than those generated from ground data, which are larger than AGB estimated by Navarro et al. [65], who found that the AGB estimated by UAV-SfM is only 10-20% less than that estimated with the ground data. However, our result is still reliable and comparable to results obtained using LiDAR systems, which have an *R*^2^ of 0.702 for AGB but more expensive [66].

The genetic gain of AGB and BGB both generated from UAV and ground truth data from two sites yields a similar result with different selection ratio (Figure 9) highlighting that the UAV-SfM technology can be suitable for tree breeding selection.

In the literature, more attention has been paid to the aboveground biomass and less to the belowground biomass analysis. Under the optimal portioning hypothesis mentioned by Bloom et al. [67] and Chapin et al. [68], plants allocate biomass in different parts of the plant under different environmental conditions. This is to maximize their ability to store water, light, and nutrients obtained for optimal growth rate. More biomass is gained in the root system in low nutrient or low water conditions but more biomass is gained in the leaves when high nutrient or high water conditions occurs [69]. The belowground biomass is as important as the aboveground biomass, and both are the central issues in plant ecology [70].

The tree BGB is usually measured through the roots and shoots. Due to the intrinsic difficulty of measuring the root system, BGB can be predicted by tree height and DBH [71]. For instance, Varik et al. [72] successfully used only DBH to predict the coarse root biomass in silver birch (*Betula pendula* Roth) (*R*^2^ = 0.89), which is higher than our results. However, the methods that they adopted was not only cost- and time-consuming, but also destructive sampling which is not suitable for high-throughput and rapid estimation. Furthermore, BGB estimation in different tree species model accuracies may vary. Here, we used the slash pine biomass allometric equation provided by Fu et al. [15] and yielded a promising result for further analysis. To the best of our knowledge, there is limited literature that assesses BGB by using the UAV-SfM method, which has not been used for breeding selection.

### 4.2. Genetic Variation in Tree Growth Traits and Biomass Yield

Determining genetic variability in growth trait yield by using UAV technology has been reported previously. Solvin et al. [73] reported that the UAV-estimated tree height is sufficient for breeding purposes in Norway spruce (*Picea abies* L. Karst.; *h*^2^ = 0.19 ~ 0.28). Similarly, Liziniewicz et al. [74] reported a broad-sense estimate of heritability ranging from 0.21 to 0.30 for tree height and DBH at different ages of Norway spruce using UAV technology. In our study, instead, *h*^2^ on tree growth traits is relatively higher (from 0.13 to 0.47). Few studies have used UAV technology to estimate biomass genetic variation. Our results showed that relatively low *h*^2^ values of AGB and BGB that ranged from 0.14 to 0.15 were found at site 1, and moderate *h*^2^ values ranging from 0.32 to 0.33 were found at site 2, which are lower than the values reported by Aranda et al. [75]. They found that tree AGB and BGB have high narrow-sense heritability values ranging from 0.77 to 0.99 when using traditional methods in *Pinus pinaster* Ait. populations.

In our study, the site influences have occurred because *h*^2^ values at site 1 are all lower than those at site 2 (in a small gully), different to the study reported by Li et al. [76] and Berlin et al. [77], who reported that the growth traits were highly influenced by the *G* × *E* interaction. The location is also an important factor that largely favored genetic gains for growth and biomass traits. Altitude and unbalanced tree numbers in these two sites cause the difference in genetic gain. However, despite the differences in sites, there was no *G* × *E* interaction in this study.

Heritability is overestimated in controlled environments compared with natural conditions [78]. Regular estimation of heritability of half-sibling tree families uses 1/4 as relationship coefficient. Here, we used a relationship coefficient of 1/2.5 to estimate the heritability of half-sibling slash pine families (Equations (6)), leading our *h*^2^ values to be lower than the *h*^2^ values of previous studies. Due to the limited information on the population structure and reproductive biology of slash pine, the genetic structure of the pine population is complex (Xiao-Fei et al. [79]). However, the moderate *h*^2^ and relatively consistent within families in our sites (Figure 7) showed that breeding selection for tree growth traits and biomass yield maybe possible and reliable. Based on the breeding values, families with high AGB and BGB coupled with optimal growth traits were selected for breeding targets. In our case, the ground truth of AGB and BGB yields a similar genetic gain with the UAV generated AGB and BGB at different selection ratio in both sites, indicating that, for breeding selection, the use of the data from UAV technology may be possible and reliable.

## 5. Conclusions

In this paper, we showed that a low-cost UAV-SfM method is a promising, accurate, and high-throughput method for assessing the growth traits, aboveground and belowground biomass of slash pine for breeding purposes. The tree height and CA metrics generated from UAV-SfM data had a high correlation with the ground truth data. The heritable variations in growth traits, AGB and BGB were significant, and the optimal families were selected for further breeding use. This method debuts the possibility of repeatable UAV surveys by providing a faster and cost-effective approach for monitoring tree growth monthly or annually over larger areas and enhances breeding programs in comparison to traditional ground surveys.

## Figures and Tables

**Table 1:** UAV parameter settings.

UAV flight information			
UAV flying altitude (m)	35	45	70
Flying time (min)	45.35	42.47	17.56
No. of images	1156	926	469
Point cloud density (points/m^2^)	435.83	254.77	99.2
Ground sampling distance (cm)	1.37	1.62	2.74
Velocity (m/s)	3.9	3.9	2.0
Shooting mode	Timed	Timed	Timed

**Table 2:** The results of multiple UAV flight altitudes (35, 45, and 70 m) based on the standard deviation between the collected tree height, DBH, CA, AGB, and BGB. Significant differences (*α* = 0.05) were assessed for collection information using Tukey's HSD test. Values inside the parenthesis are standard errors.

	Height (m)	DBH (cm)	CA (m^2^)	AGB (kg)	BGB (kg)
Flying altitude (m)					
35	7.67 (±1.22)a	18.80 (±4.70)a	13.86 (±3.86)a	101.42 (±73.80)a	33.95 (±27.06)a
45	7.76 (±1.21)a	18.95 (±4.72)a	13.83 (±3.83)a	103.53 (±74.56)a	34.58 (±27.27)a
70	7.75 (±1.24)a	18.93 (±4.79)a	13.79 (±3.85)a	103.67 (±77.23)a	34.64 (±28.30)a

**Table 3:** Genetic correlations (below diagonal) between traits at the two sites, with standard errors shown in parentheses.

Traits	UAV_AGB	UAV_BGB	UAV_CA	UAV_H	PRE_DBH	GT_AGB
*Site 1*						
UAV_BGB	1.00 (±0.00)					
UAV_CA	0.61 (±0.24)	0.59 (±0.25)				
UAV_H	0.99 (±0.06)	0.99 (±0.07)	0.77 (±0.18)			
PRE_DBH	0.97 (±0.02)	0.96 (±0.03)	0.78 (±0.15)	1 (±0.03)		
GT_AGB	0.71 (±0.13)	0.69 (±0.14)	0.67 (±0.15)	0.76 (±0.11)	0.73 (±0.12)	
GT_BGB	0.62 (±0.16)	0.60 (±0.13)	0.61 (±0.16)	0.67 (±0.14)	0.64 (±0.15)	0.99 (±0.01)
*Site 2*						
UAV_BGB	1.00 (±0.00)					
UAV_CA	0.9 (±0.07)	0.90 (±0.07)				
UAV_H	0.79 (±0.11)	0.75 (±0.13)	0.68 (±0.15)			
PRE_DBH	0.98 (±0.01)	0.96 (±0.02)	0.92 (±0.05)	0.87 (±0.07)		
UAV_R	0.89 (±0.07)	0.89 (±0.08)	1.00 (±0.00)	0.69 (±0.15)	0.92 (±0.05)	
GT_AGB	0.93 (±0.06)	0.93 (±0.06)	0.84 (±0.13)	0.86 (±0.10)	0.94 (±0.05)	
GT_BGB	0.90 (±0.08)	0.91 (±0.07)	0.88 (±0.11)	0.81 (±0.14)	0.92 (±0.07)	0.99 (±0.01)

## Data Availability

The data mentioned in this study are available on request from the corresponding author.

## Supplementary Materials

Supplementary 1 Figure S1: The cloud point data of one example tree generated from three different flight altitudes.

Supplementary 2 Figure S2: The linear model and the ground truth data, UAV data (height, AGB, BGB) and predicted DBH at 35 m and 45m flying heights.
