# Supplementary figures and images for: Enabling Breeding Selection for Biomass in Slash Pine Using UAV-Based Imaging
Source: Plant Phenomics. 2022 Apr 22;2022:9783785. doi: 10.34133/2022/9783785 (PMC9057123; doi:10.34133/2022/9783785)

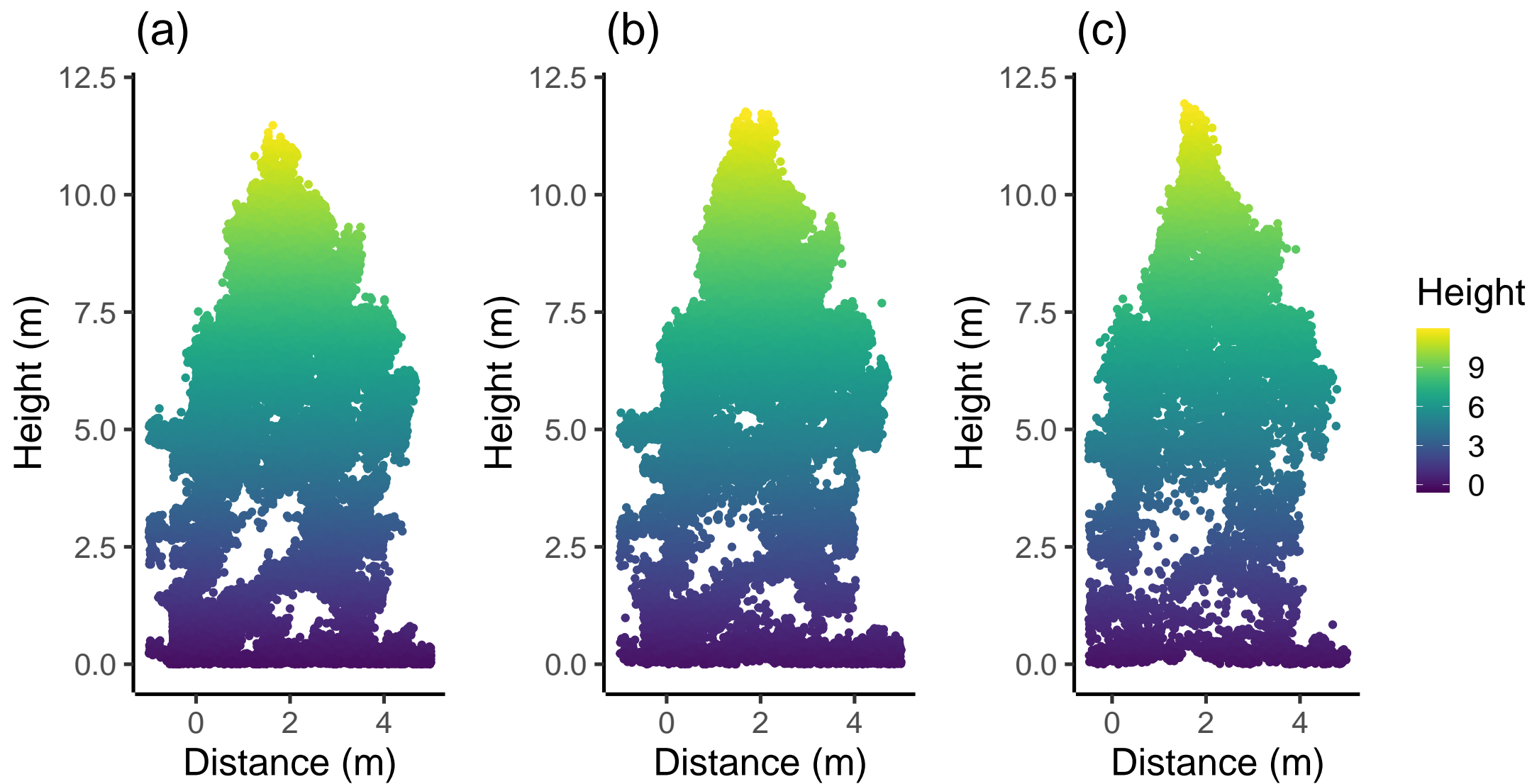

Supplement: Supplementary 1 — Figure S1: The cloud point data of one example tree generated from three different flight altitudes. [file 9783785.f1.pdf]

35m

45m

Height(m)

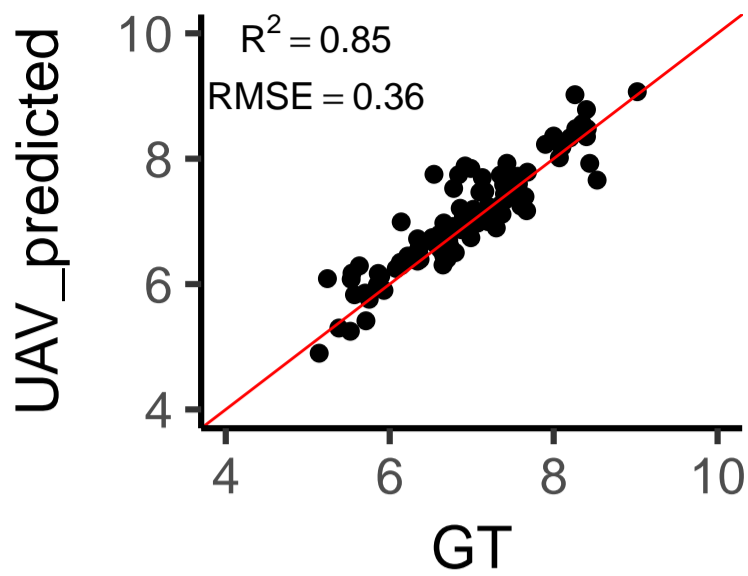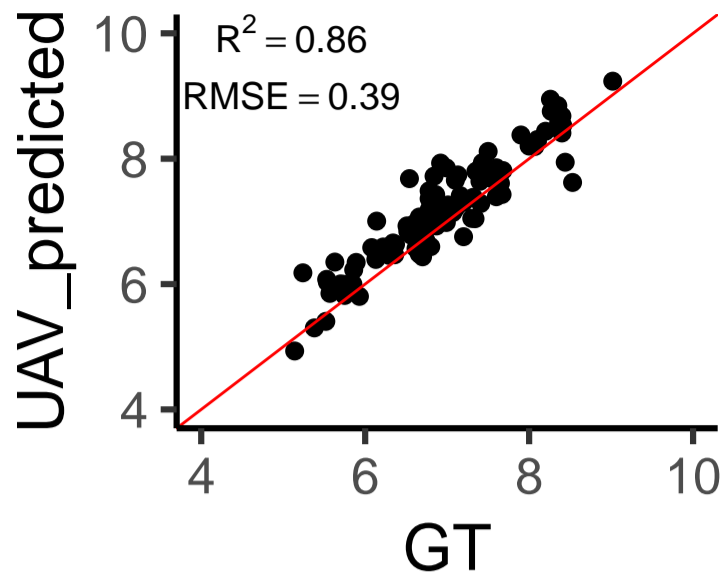

DBH(cm)

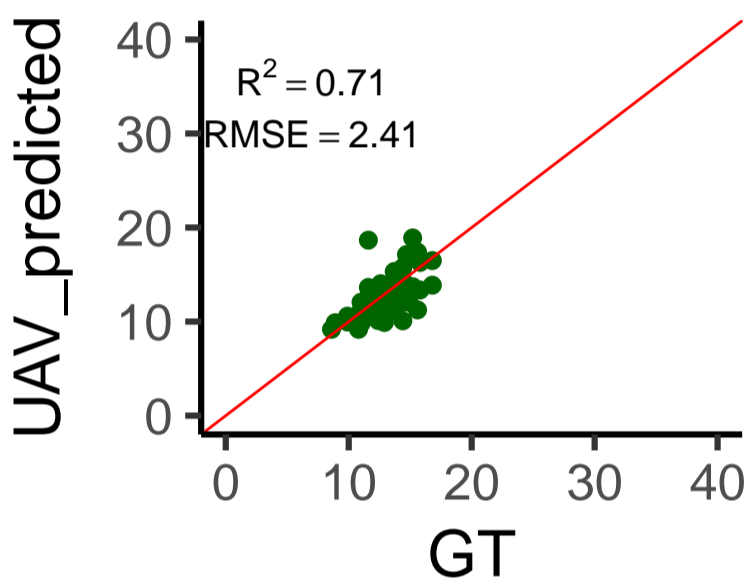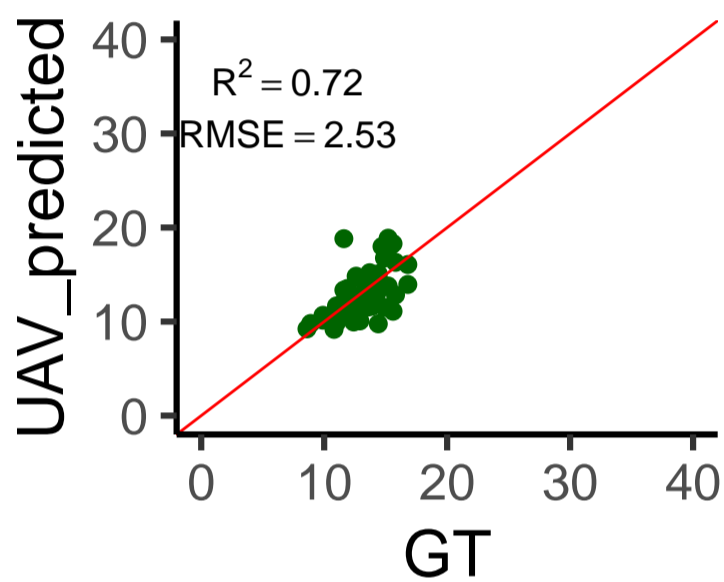

AGB(kg)

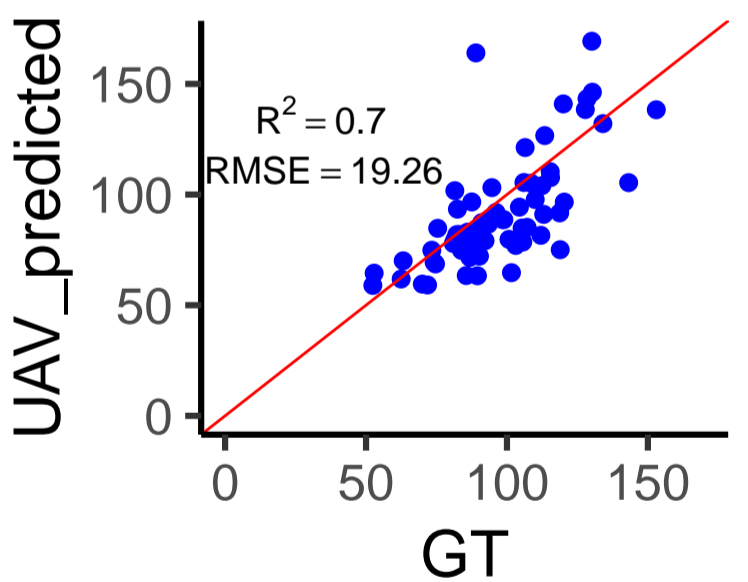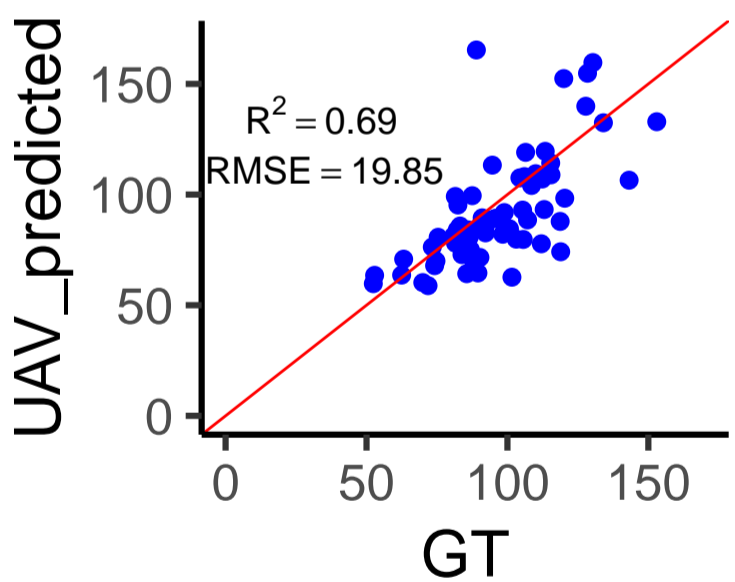

BGB(kg)

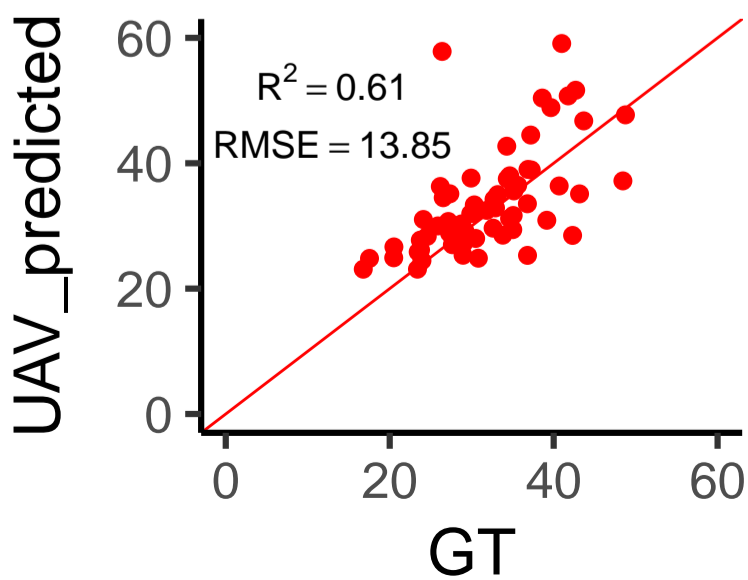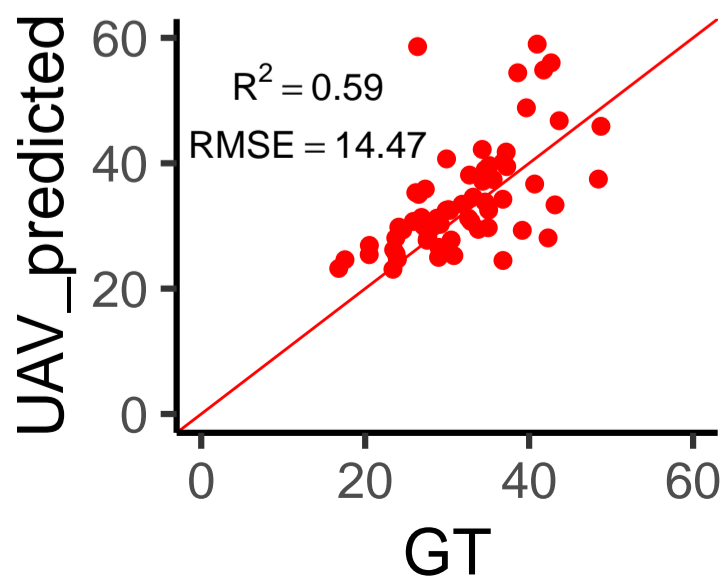

Supplement: Supplementary 2 — Figure S2: The linear model and the ground truth data, UAV data (height, AGB, BGB) and predicted DBH at 35 m and 45m flying heights. [file 9783785.f2.pdf]
